# Physiological, Biochemical and Molecular Response of Different Winter Wheat Varieties under Drought Stress at Germination and Seedling Growth Stage

**DOI:** 10.3390/antiox11040693

**Published:** 2022-03-31

**Authors:** Rosemary Vuković, Ivna Štolfa Čamagajevac, Ana Vuković, Katarina Šunić, Lidija Begović, Selma Mlinarić, Ramona Sekulić, Nikolina Sabo, Valentina Španić

**Affiliations:** 1 Department of Biology, University of Osijek, 31000 Osijek, Croatia; rosemary@biologija.unios.hr (R.V.); ivna@biologija.unios.hr (I.Š.Č.); ana.vukovic@biologija.unios.hr (A.V.); lidija.begovic@biologija.unios.hr (L.B.); selma.mlinaric@biologija.unios.hr (S.M.); ramona.sekulic@biologija.unios.hr (R.S.); nsabo@biologija.unios.hr (N.S.); 2 Department of Small Cereal Crops, Agricultural Institute Osijek, 31000 Osijek, Croatia; katarina.sunic@poljinos.hr

**Keywords:** antioxidative response, dehydrins, drought, osmotic adjustment, transcription factors, wheat

## Abstract

Due to climate change in recent years, there has been an increasing water deficit during the winter wheat sowing period. This study evaluated six Croatian winter wheat varieties’ physiological, biochemical, and molecular responses under two drought stress levels at the germination/seedling growth stage. Lipid peroxidation was mainly induced under both drought stress treatments, while the antioxidative response was variety-specific. The most significant role in the antioxidative response had glutathione along with the ascorbate-glutathione pathway. Under drought stress, wheat seedlings responded in proline accumulation that was correlated with the *P5CS* gene expression. Expression of genes encoding dehydrins (*DHN5*, *WZY2*) was highly induced under the drought stress in all varieties, while genes encoding transcription factors were differentially regulated. Expression of *DREB1* was upregulated under severe drought stress in most varieties, while the expression of *WRKY2* was downregulated or revealed control levels. Different mechanisms were shown to contribute to the drought tolerance in different varieties, which was mainly associated with osmotic adjustment and dehydrins expression. Identifying different mechanisms in drought stress response would advance our understanding of the complex strategies contributing to wheat tolerance to drought in the early growth stage and could contribute to variety selection useful for developing new drought-tolerant varieties.

## 1. Introduction

Due to climate change, years with more extended periods of water deficit and heat stress are becoming more frequent and, thus, threatening global crop production [1,2]. Drought conditions particularly affect the yield and quality of wheat (*Triticum aestivum* L.), one of the most important and widespread grain crops, indispensable in human nutrition and animal feed production [3]. Although the region of Croatia is characterized by large seasonal variability, the increasing annual frequency of dry days was encountered all over the country in the past years [4]. In previous research, the evaluation of forty Croatian winter wheat genotypes showed a decrease in wheat yield by 14–50% under water deficit conditions [5].

The germination and seedling growth stage is one of the most sensitive stages in plant development, implying the importance of the plant’s tolerance to drought in the early growth stage [6,7]. In winter wheat, water deficit during the sowing period affects seed germination and causes a change in physiological and biochemical processes during seedling establishment, resulting in reduced growth, development, and overall productivity [8,9]. Osmotic stress caused by salinity and drought has a significant impact on plant productivity as a result of water limitations [10]. In response to osmotic stress, plants have adopted various drought tolerance mechanisms, including the formation of deeper roots, increased biomass, increased antioxidative metabolism, accumulation of osmoprotectants to facilitate osmotic adjustment, expression of different stress-responsive genes, and others [11,12,13,14,15].

Drought conditions in plant seedlings induce reactive oxygen species (ROS) production, which, in low concentrations, have a signaling role in abiotic stress response pathways. In contrast, higher ROS concentrations cause oxidative damage to the cellular biomolecules, such as proteins, lipids, and nucleic acids [16,17]. Lipid peroxidation induced by ROS disrupts membrane structure, leading to loss of its selectivity and integrity as well as disruption of water balance and nutrient uptake. Such imbalance can have detrimental effects on photosynthesis essential for biomass accumulation and therefore shoot and root elongation [18]. To maintain a balance between ROS production and scavenging, plants have developed an antioxidant defense system that includes nonenzymatic antioxidants and antioxidant enzymes such as superoxide dismutase (SOD), catalase (CAT), and the enzymes of the ascorbate-glutathione (AsA-GSH) pathway [19]. The AsA-GSH pathway is essential in detoxifying ROS and interacts with other defense systems to mitigate oxidative damage induced by abiotic stress [20]. This pathway entails the two potent antioxidants, glutathione and ascorbic acid, and four enzymes, namely ascorbate peroxidase (APX), monodehydroascorbate reductase (MDHAR), dehydroascorbate reductase (DHAR), and glutathione reductase (GR), that contribute to the maintenance of cellular glutathione and ascorbic acid pools in the plant cell, thus protecting plants from abiotic stress [20,21]. Glutathione is one of the most common nonenzymatic antioxidants, essential in maintaining the stability of the redox state in all plant cell compartments [22]. Glutathione has a role in plant growth and development as well as in defense mechanisms against various environmental stresses. It can directly neutralize ROS or indirectly act as a substrate of different enzymes for ROS and toxic substances removal [23,24]. Therefore. plants’ stress tolerance is extensively associated with the glutathione redox state maintained by the AsA-GSH pathway [20,21].

Under water-deficit conditions, plants have developed osmotic adjustment, one of the fundamental biochemical mechanisms of drought adaptation [14]. Osmotic adjustment changes cell osmotic potential due to the accumulation of osmolytes, thus preserving the physiological functions of the cell in stressful conditions. In addition to their role in osmoregulation, osmolytes have a role in maintaining membrane and other subcellular structures, in reactive oxygen species scavenging, and in gene expression regulation [25,26]. Depending on the plant metabolism, developmental stage, and environmental conditions, different plants species can accumulate different osmolites such as amino acids, sugar alcohols, sugars, quaternary amines, and other low-molecular-weight organic solutes [27,28]. Previous studies showed that wheat’s proline accumulation contributes to increased osmotic stress tolerance [29,30]. In plants, there are two pathways for proline biosynthesis, and the preferred one includes the conversion of glutamate to proline by two successive reactions catalyzed by the enzymes Δ^1^-pyrroline-5-carboxylate synthetase (P5CS) and Δ^1^-pyrroline-5-carboxylate reductase [31,32]. The expression of the *P5CS* gene encoding the P5CS enzyme in wheat was shown to be upregulated under osmotic stress and correlated with proline accumulation [29]. Moreover, overexpression of the *P5CS* gene in transgenic wheat resulted in increased stress tolerance to water-deficit conditions due to increased proline content [33].

One of the strategies to combat water-deficit conditions is an expression of protective proteins like dehydrins, an important group of late embryogenesis abundant proteins. Accumulation of dehydrins is induced by different developmental stages and by different abiotic stress factors. Several studies reported a positive correlation between the accumulation of dehydrin transcripts or proteins and drought tolerance [34,35]. Overexpression of wheat dehydrin gene (*DHN5*) enhances tolerance to salt and osmotic stress in *Arabidopsis* plants due to regulation of proline metabolism and antioxidative response [36,37,38].

In plants’ stress tolerance, stress recognition and signal transduction are crucial in inducing adequate cell response and in the regulation of stress-related genes [25,39]. In abiotic stress signal transduction, transcription factors are terminal transducers that directly regulate the expression of downstream stress-responsive genes by interacting with their promotor region [39]. The most characterized transcription factor families involved in plant abiotic stress are WRKY and AP2/EREBP family DREBs (dehydration-responsive element-binding proteins). Both transcription factor families regulate developmental, physiological, and metabolic processes [40,41]. WRKY transcription factors are defined by the WRKY domain, composed of a highly conserved WRKYGQK sequence important for protein–protein interaction, followed by a zinc-finger motif with DNA binding affinity. Based on the number of the WRKY domains and the type of zinc-finger motif, these transcription factors can be classified into three main groups (I, II, and III) [42,43]. The DREB subfamily is defined by the presence of a single highly conserved AP2/ethylene-responsive element-binding factor (ERF) DNA binding domain that specifically binds to dehydration-responsive element (DRE)/C-repeat element (CRT) cis-elements at the promoter of the dehydration/cold-regulated (RD/COR) genes, responsive to water deficit and low temperature [44,45]. Previous studies showed that many genes encoding different DREB and WRKY transcription factors in wheat are upregulated under exposure to water-deficit conditions, thereby improving the tendency of wheat to tolerate drought stress [46,47,48,49]. Some of the genes encoding DREB and WRKY transcription factors have been used in transgenic technology to improve stress tolerance in model and crop plants [40]. Overexpression of these genes in transgenic plants showed enhanced tolerance to multiple abiotic stresses [50,51,52,53]. Transgenic wheat seedlings overexpressing the *TaWRKY2* gene exhibited enhanced tolerance to drought stress [52]. Overexpression of *TaWRKY2* in transgenic *Arabidopsis* plants exhibited salt and drought tolerance, while overexpression of *TaWRKY19* conferred tolerance to salt, drought, and freezing stresses in transgenic plants [54]. Transgenic *Arabidopsis* plants with overexpression of wheat *TaDREB3A1* gene showed enhanced tolerance against heat, drought, and salt stresses [51].

The impact of drought stress has been well documented in many crop species, but this study includes an integrated approach on morpho-physiological, biochemical, and molecular levels in six winter wheat varieties under drought stress conditions at the seedling growth stage. The study includes Croatian genotypes selected due to their importance in production and based on previous field trials, which showed different susceptibility to drought conditions. Due to different susceptibilities to drought, we assumed different winter wheat varieties’ antioxidant responses, osmotic adjustment, expression of stress-responsive genes, and genes encoding transcription factors. The obtained results will enable us to select drought-tolerant varieties in the seedling stage of growth and understand the complex mechanisms contributing to their tolerance.

## 2. Materials and Methods

### 2.1. Plant Material and Treatments

For drought-tolerance evaluation, six commonly used Croatian winter wheat varieties originated from Agricultural Institute Osijek were selected (Silvija, Rujana, Bubnjar, Fifi, Anđelka, and Pepeljuga) and subjected to different levels of drought stress in the germination and seedling stage of growth. Before germination, wheat seeds were sterilized with 70% ethanol for 1 min and washed two times in dH_2_O. For each wheat variety, 40 seeds per treatment were germinated on a filter paper in glass jars (H 9 × W 10 cm, V = 0.5 L) supplied with 10 mL of polyethylene glycol 6000 (PEG) in final concentrations of 10 and 20% for drought stress induction, while seedlings growing in water were used as a control. Wheat seedlings were grown in a growth chamber under a 16/8 h light/dark photoperiod, 25/20 °C day/night temperature, and 60% relative humidity. During the growing period, 10 mL of the solution related to each treatment was added daily to the glass jars. After seven days of growth, wheat seedlings were sampled for morpho-physiological characterization and biochemical and molecular analysis. For biochemical and molecular analysis, the tissue of seedlings was frozen in liquid nitrogen and disrupted in 10 mL stainless steel jars containing a grinding ball (Ø 20 mm) for 1 min at 30 Hz using a TissueLyser II bead mill (Qiagen). Metabolites, proteins, and RNA were extracted from the tissue powder aliquots homogenized with an appropriate extraction solution.

### 2.2. Morpho-Physiological Traits Measurements

On the fourth day of germination, sprouted seeds were counted, and the germination energy was calculated, taking into account the total number of germinated seeds and the initial number of seeds according to the formula: (number of seeds germinated on the fourth day/total number of seeds) × 100. Wheat seedlings were sampled seven days after germination, whereas the shoots and roots were separated to assess the morphological traits. Shoots and roots length was measured and expressed in mm per plant. For the biomass estimation, a fresh mass of the shoots and roots was measured, while dry mass was determined after drying wheat tissue in an oven at 105 °C for 24 h. Shoot and root biomass was expressed in terms of dry weight (DW) per plant. For relative water content (RWC) estimation, fresh weight (FW) of young leaves was determined immediately after sampling, after which leaves were soaked in the dH_2_O for 24 h for hydration. After 24 h, turgid weight (TW) was determined, and the leaves were dried in the oven at 105 °C for 24 h for dry weight (DW) estimation. RWC (%) was calculated as (FW-DW)/(TW-DW) × 100 [55]. All morpho-physiological traits were determined from 15 seedlings per experimental group.

### 2.3. Determination of the Proline Content

Proline content was determined according to Carillo and Gibon [56]. Proline was extracted from the 0.1 g frozen tissue powder in 40% ethanol overnight at 4 °C. After cold extraction, the homogenate was centrifuged for 5 min at 14,000× *g*. An aliquot of extract (0.05 mL) was incubated with 0.1 mL of a ninhydrin reagent (1% (*w/v*) ninhydrin in 60% (*v/v*) acetic acid and 20% ethanol (*v/v*)) at 95 °C for 20 min on a TS-100 Thermo-Shaker (Biosan, Riga, Latvia). After cooling and brief centrifugation, an 0.1 mL aliquot of the reaction mixture was transferred to a 96-well microplate, and the absorbance was measured at 520 nm and 25 °C using Spark multimode microplate reader with SparkControl software (Tecan, Männedorf, Switzerland). Proline content was determined using a standard curve with proline, and the results were expressed in nmol/mg FW.

### 2.4. Determination of the Lipid Peroxidation Level

Lipid peroxidation levels in wheat seedlings were estimated by measuring the thiobarbituric acid reactive substances (TBARS), according to the method described by Verma and Dubey [57]. This method is based on the formation of red pigment, generated by the reaction of lipid peroxidation breakdown products like malondialdehyde (MDA) with thiobarbituric acid at an optimum pH of 3.5. Briefly, the frozen wheat powder was homogenized with 0.1% trichloroacetic acid (TCA) solution (1/5, *w/v*) and centrifuged for 10 min at 10,000× *g* and 4 °C. The reaction mixture that consisted of 0.5 mL of tissue extract and 1 mL of reagent (0.5% thiobarbituric acid in 20% TCA) was incubated for 30 min at 95 °C on a TS-100 Thermo-Shaker (Biosan, Riga, Latvia). After cooling the reaction mixture, the produced red pigment was measured at 532 and 600 nm on a LAMBDA 25 UV-Vis spectrophotometer equipped with UV WinLab v6.0.4 software package (PerkinElmer, Waltham, MA, USA). The results were expressed as nmol/g of FW.

### 2.5. Determination of the Glutathione Content

Total (tGSH) and oxidized glutathione (GSSG) content were determined using a kinetic method based on a continuous reduction of 5,5-dithiobis (2-nitrobenzoic acid) (DTNB) to 5-thio-2-nitrobenzoic acid (TNB) by reduced glutathione (GSH), where NADPH reduces the GSSG in the presence of GR [58]. The method is modified for the microplate assay, and the measurements were performed using Greiner UV Star 96-well plates on a Spark multimode microplate reader with SparkControl software (Tecan, Männedorf, Switzerland). For glutathione content determination, the frozen wheat powder was homogenized with 5% 5-sulfosalicylic acid solution (1/10, *w/v*) and centrifuged for 10 min at 16,000× *g* and 4 °C. Reaction mixture consisted of 0.031 mg/mL DTNB, 0.115 U/mL of GR, 1 mM EDTA, and 10 μL of deproteinized extract in 100 mM phosphate buffer (pH 7.0), in a final volume of 0.21 mL. After 5 min of equilibration, the reaction was initiated by adding NADPH at a final concentration of 48 μM. The formation of TNB was continuously recorded at 412 nm for 5 min every 15 s, at 25 °C. The amount of tGSH was determined using a standard curve of GSH, and the results were expressed as nmol/g of FW. For GSSG determination, aliquots of deproteinized extracts were incubated with vinylpyridine and triethanolamine for one hour at room temperature for GSH removal. The measurements were performed in the same way as for the tGSH. The content of GSSG was determined using a standard GSSG curve, and the results were expressed in nmol/g of FW. The amount of GSH was obtained from the difference between tGSH and GSSG and expressed in nmol/g of FW.

### 2.6. Antioxidant Enzymes Activity Determination

Protein extracts were prepared by homogenizing an aliquot of frozen wheat powder with a 100 mM phosphate buffer (pH 7.0) containing 1 mM EDTA (1/5, *w/v*). Proteins were extracted after 15 min of incubation on ice and centrifugation at 20,000× *g* for 15 min at 4 °C. Protein extracts were stored at −80 °C until further analysis. The enzymes’ activities were measured at 25 °C using a LAMBDA 25 UV-Vis spectrophotometer equipped with UV WinLab v6.0.4 software package (PerkinElmer, Waltham, MA, USA) and Spark multimode microplate reader with SparkControl software (Tecan, Männedorf, Switzerland).

CAT (EC 1.11.1.6) activity was estimated according to the method described by Aebi [59] using H_2_O_2_ as a substrate. The reaction mixture consisted of 0.036% H_2_O_2_ in 50 mM phosphate buffer pH (7.0), and the reaction started with the addition of 50 μL of diluted protein extract. The decrease in absorbance due to the oxidation of H_2_O_2_ was measured at 240 nm for 3 min every 10 s. CAT activity was calculated using molar extinction coefficient (ε = 0.04 mM/cm) and expressed as U/mg of protein.

GST (EC 2.5.1.13) activity was determined by the method of Habig et al. [60], which is based on the formation of glutathione-2,4-dinitrobenzene (G-SDNB) due to the conjugation of 1-chloro-2,4-dinitrobenzene (CDNB) with GSH. The reaction mixture consisted of 2 mM GSH, 1 mM CDNB, 1 mM EDTA, and 50 μL of protein extract in 100 mM phosphate buffer (pH 6.5), in a final volume of 1.5 mL. The increase in absorbance was recorded at 340 nm for 2 min every 15 s. GST activity was calculated using a molar extinction coefficient of glutathione-1-chloro-2,4-dinitrobenzene conjugate (ε = 9.6 mM/cm) and expressed as U/g of protein.

APX (EC 1.11.1.11) activity was determined according to the method described by Nakano and Asada [61]. The reaction mixture consisted of 0.5 mM ascorbic acid, 0.12 mM H_2_O_2_, 0.1 mM EDTA and 50 μL of diluted protein extract in 50 mM potassium phosphate buffer (pH 7.0), in a final concentration of 1 mL. The decrease in absorbance was measured at 290 nm for 2 min every 10 s. The APX activity was calculated using a molar extinction coefficient (*ε* = 2.8 mM/cm) and expressed in U/mg of protein.

GR (EC 1.6.4.2) activity was determined according to the method described by Racker [62], which is based on measuring NADPH during the reduction of GSSG. The reaction mixture consisted of 1 mM GSSG, 1 mM EDTA, and 25 μL of protein extract in 100 mM phosphate buffer (pH 7.5), in a final volume of 1 mL. After 10 min of equilibration at 25 °C, the reaction was started by adding NADPH in a final concentration of 0.1 mM. The decrease in absorbance was monitored at 340 nm for 2 min every 15 s. GR activity was calculated using the molar extinction coefficient for NADPH (ε = 6.22 mM/cm) and expressed in U/g protein.

DHAR (EC 1.8.5.1) activity was determined according to the method described by Ma and Cheng [63] and modified for microplate assay by Murshed et al. [64]. The method is based on glutathione-dependent reduction of dehydroascorbate (DHA). The reaction mixture consisted of 0.1 mM EDTA, 2.5 mM GSH, 0.2 mM DHA, and 10 μL of protein extract in 50 mm HEPES buffer (pH 7.0), in a final volume of 0.2 mL. The increase in absorbance was monitored at 265 nm for 3 min every 15 s. DHAR activity was calculated using molar extinction coefficient (ε = 8.33 mM/cm) and expressed in U/g protein.

The activity of MDHAR (EC 1.6.5.4) was determined by the method described by Hossain et al. [65] with modifications for the microplate assay. The method is based on reducing mono-dehydroascorbate, generated by the ascorbate oxidase, to ascorbate using NADH as the reducing agent. The reaction mixture comprised 2.5 mM ascorbate, 0.1 mM NADH, 0.14 U of ascorbate oxidase, and 10 μL of protein extract in 50 mM Tris-HCl buffer (pH 7.6), with a final volume of 0.2 mL. The decrease in absorbance was recorded at 340 nm for 3 min every 15 s. MDHAR activity was calculated using molar extinction coefficient (ε = 3.7 mM/cm) and expressed in U/g protein.

Total protein concentration in tissue extracts was estimated by the method of Bradford [66], using bovine serum albumin, ranging from 0.1 to 1.4 mg/mL, as a standard. For protein determination, a microwell plate assay was used where 5 μL of diluted protein extracts was added to 250 µL of Bradford reagent (Sigma-Aldrich, St. Louis, MI, USA), and, after a five-minute incubation, the sample was read at 595 nm on a Spark multimode microplate reader with SparkControl software (Tecan, Männedorf, Switzerland).

### 2.7. RNA Isolation, cDNA Synthesis, and Quantitative PCR

Total RNA was isolated from 50 mg of frozen wheat tissue powder using the NucleoZOL reagent (Macherey-Nagel), following the manufacturer’s instructions. Subsequently, residual DNA in the obtained RNA solution was removed by rDNase (Macherey-Nagel). For DNA digestion, 1/10 volume of the rDNase-buffer premix (1/10, *v/v*) was added to the RNA solution and incubated for 10 min at 37 °C. RNA was subsequently repurified by ethanol precipitation: 0.1 volume of 3 M sodium acetate (pH 5.2) and 2.5 volumes of 100% ethanol were added to one sample volume. After two hours of incubation at −20 °C, samples were centrifuged for 10 min at maximum speed. The RNA pellet was washed with 70% ethanol, dried, and resuspended in RNase-free water. RNA concentrations and purity were assessed using NanoPhotometer NP-80 (Implen, München, Germany). The average RNA yield was around 1000 ng/μL, while A260/A280 ratio was approximately 2.0. RNA integrity was verified on agarose gel electrophoresis and visualized by SYBR safe staining (Invitrogen).

First-strand cDNA was synthesized from 3 μg of total RNA using the GoTaq^®^ 2-Step RT-qPCR System (Promega) according to the manufacturer’s recommendation. After denaturation of the RNA template and Oligo(dT)_15_ primer premix at 70 °C for 5 min, the cDNA was synthesized in a final volume of 20 μL by combining the denatured premix with the reaction mixture consisting of 1× GoScript buffer, 2.5 mM MgCl_2_, 0.5 mM nucleotide mix, 20 U of ribonuclease inhibitor, and 1U of reverse transcriptase. The cDNA synthesis was performed under the following conditions: primer annealing at 25 °C for 5 min, extension at 42 °C for 1 h, and enzyme inactivation at 70 °C for 5 min. All incubation steps were performed on the MiniAmp Plus Thermal PCR Cycler (Applied Biosystems, Waltham, MA, USA).

Following cDNA synthesis, quantitative PCR (QPCR) using dye-based detection was performed to analyze transcript levels of seven genes (*P5CS*, *DHN5*, *WZY2*, *DREB1*, *WRKY2*, *actin,* and *ADP ribosylation factor*). The specific oligonucleotide primers were designed based on sequences in the GeneBank database using Primer3 software (Table 1). Some primers were designed to span the exon–exon junction containing an intron to differentiate between RNA versus genomic DNA amplification, thus confirming the absence of DNA contamination. qPCR analysis was performed on StepOnePlus™ Real-Time PCR System with StepOnePlus™ Software v2.3 (Applied Biosystems, Waltham, MA, USA) and by using GoTaq^®^ 2-Step RT-qPCR System (Promega), according to the manufacturer’s recommendation. The qPCR amplification of all target sequences was performed in a 25 μL reaction mixture containing 5 μL of five-fold diluted cDNA template, 12.5 μL of GoTaq qPCR Master Mix (2×), 200 nmol of each primer, and 0.25 μL CXR reference dye. The qPCR amplification was performed under the following conditions: GoTaq Hot Start Polymerase activation at 95 °C for 2 min, followed by 40 cycles consisting of denaturation at 95 °C for 15 s, primer annealing, and extension at 60 °C for 1 min. The specificity of the QPCR reaction was confirmed by melting curve analysis. Three biological replicates were used in quantification analysis, and three technical replicates were analyzed for each biological replicate. Relative gene expression was quantified using a relative standard curve based on five points, corresponding to a three-fold dilution series from pooled cDNA, and normalized using the geometric average of two reference genes, *actin* and *ADP-ribosylation factor*.

### 2.8. Data Analysis

Data were analyzed using the statistical program GraphPad Prism 5.03. The data were presented as the mean of six (or three for gene expression analysis) replicas ± standard deviations (SD). Differences among treatments in each variety separately were assessed by a one-way analysis of variance (ANOVA), followed by the Dunnett post hoc test. The Dunnett test was performed at a significance level of 5, 1, and 0.1% (* *p* < 0.05, ** *p* < 0.01, *** *p* < 0.001).

## 3. Results

### 3.1. Morpho-Physiological Traits

**Figure 1:**
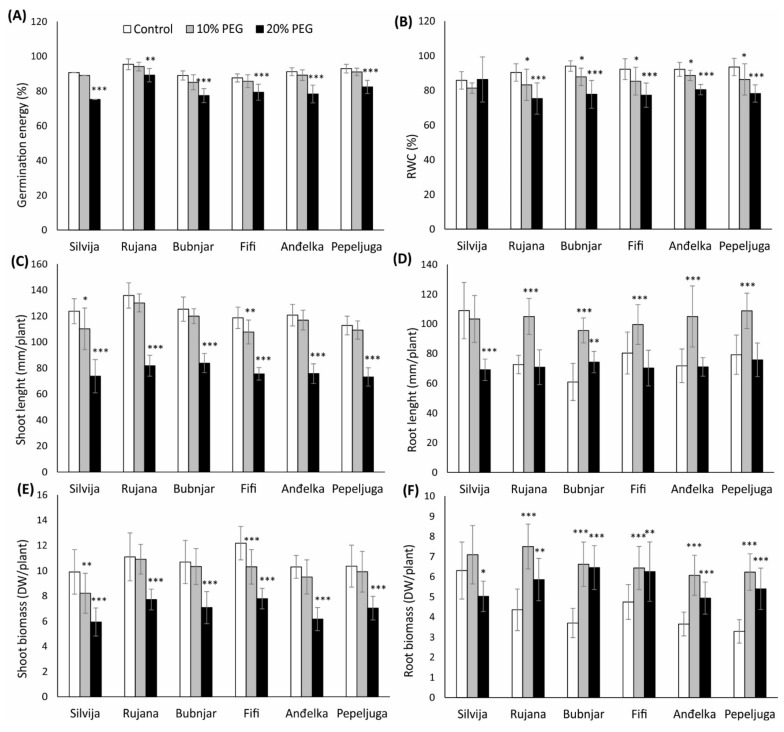
Morpho-physiological traits: (**A**) germination energy, (**B**) relative water content (RWC), (**C**) shoot length; (**D**) root length; (**E**) shoot biomass, and (**F**) root biomass of seedlings of six Croatian wheat varieties (Silvija, Rujana, Bubnjar, Fifi, Anđelka, and Pepeljuga) under 10 and 20% PEG treatment. Expression data are presented as means of three independent biological replicates, and the error bars indicate standard deviations. Differences among treatments in each variety separately were assessed by a one-way analysis of variance (ANOVA), followed by the Dunnett post hoc test. The Dunnett test was performed at a significance level of 5, 1 and 0.1% (* *p* < 0.05, ** *p* < 0.01, *** *p* < 0.001).

In all varieties, drought stress induced with 10% PEG did not significantly impact the grain germination, while treatment with 20% PEG significantly reduced the germination energy of all wheat varieties tested (Figure 1A). The reduction of germination energy ranged from 6.6 (*p* < 0.01) for the variety Rujana to 17% (*p* < 0.001) for the variety Silvija.

Both levels of PEG treatments significantly reduced the RWC in almost all varieties in a concentration-dependent manner, with the exception of the variety Silvija, where no significant difference occurred in both treatments compared to the control (Figure 1B). RWC reduction under 10% PEG treatment ranged from 4 to 8% (*p* < 0.05) and under the 20% PEG treatment from 13 to 17% (*p* < 0.001).

Treatment under 20% PEG significantly reduced shoot length of all varieties (*p* < 0.001), while treatment with 10% PEG reduced the shoot length only in varieties Silvija and Fifi (Figure 1C). The highest shoot length reduction (11 and 40% under 10 and 20% PEG, respectively) was shown for variety Silvija, followed by variety Fifi (9 and 36% under 10 and 20% PEG, respectively). The lowest reduction of 33% under the 20% PEG had variety Bubnjar, followed by Pepeljuga and Anđelka, relative to the control.

Treatment under 10% PEG significantly induced root growth in almost all varieties (*p* < 0.001), with the exception of variety Silvija (Figure 1D). The increase in root length ranged from the highest 57% recorded for the variety Bubnjar, followed by the variety Anđelka (46%), to the lowest significant increase detected in the variety Fifi (24%), relative to the control. Additionally, the root length of the variety Bubnjar was also increased (22%, *p* < 0.01) under the 20% PEG treatment compared to the control. Only variety Silvija showed a significant reduction in root length (36%, *p* < 0.001) under the 20% PEG treatment.

Biomass of the shoots was significantly reduced in all varieties under treatment with 20% PEG (*p* < 0.001), while the treatment under 10% PEG significantly reduced biomass only of the variety Silvija and Fifi, compared to the control (Figure 1E). Thus, the highest reduction in shoot biomass was shown for the varieties Silvija, 17 (*p* < 0.01) and 40% (*p* < 0.001) under the 10 and 20% PEG, respectively, and variety Fifi, 15 (*p* < 0.001) and 36% (*p* < 0.001) under the 10 and 20% PEG-treatment, respectively, relative to the control.

Roots biomass increased in almost all varieties under both PEG treatments, with the exception of the variety Silvija, where 20% PEG significantly reduced the root biomass by 20% (*p* < 0.05) relative to the control seedlings (Figure 1F). Treatment with 10% PEG induced an increase in root biomass, ranging from 36 to 90%, while treatment under 20% PEG increased root biomass ranging from 32 to 74%. The highest significant increase was detected in varieties Bubnjar (80 and 74% under 10 and 20% PEG, respectively) and Pepeljuga (90 and 64% under 10 and 20% PEG, respectively), while the lowest significant increase was detected in variety Fifi (36 and 32% under the 10 and 20% PEG, respectively), relative to the control.

### 3.2. Proline Content in Wheat Seedlings

**Figure 2:**
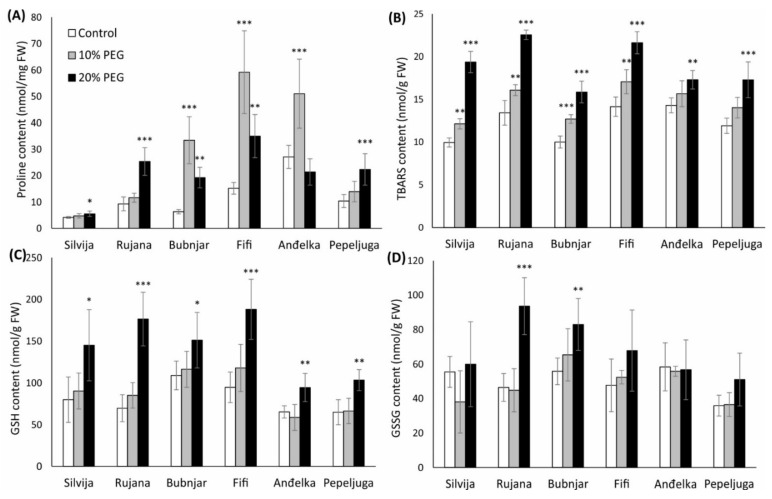
Content of (**A**) thiobarbituric reactive substances (TBARS); (**B**) proline; (**C**) reduced glutathione (GSH); and (**D**) oxidized glutathione (GSSG) in wheat seedlings of six Croatian wheat varieties (Silvija, Rujana, Bubnjar, Fifi, Anđelka, and Pepeljuga) under 10 and 20% PEG treatment. Expression data are presented as means of three independent biological replicates, and the error bars indicate standard deviations. Differences among treatments in each variety separately were assessed by a one-way analysis of variance (ANOVA), followed by the Dunnett post hoc test. The Dunnett test was performed at a significance level of 5, 1 and 0.1% (* *p* < 0.05, ** *p* < 0.01, *** *p* < 0.001).

Treatment with 20% PEG significantly increased proline content in wheat seedlings of almost all varieties, except for variety Anđelka which showed no changes relative to the control (Figure 2A). Varieties Bubnjar, Fifi, and Anđelka showed a higher proline content after the treatment with 10% PEG (*p* < 0.001) than the 20% treatment. The highest increase of proline content was recorded in variety Bubnjar, with a 4-fold increase under 10% and a 2-fold increase under 20% PEG, relative to the control. Variety Silvija showed the lowest increase in proline content under severe stress, only 33% relative to the control (*p* < 0.05).

### 3.3. Lipid Peroxidation Levels in Wheat Seedlings

Treatments with PEG increased lipid peroxidation in seedlings of all wheat varieties in a concentration-dependent manner (Figure 2B). Treatment with 10% PEG caused a significant increase in TBARS content in the varieties Bubnjar (27%), Silvija (22%), Fifi (21%), and Rujana (20%), while no significant increase was recorded for the varieties Anđelka and Pepeljuga, compared to the control. Treatment with 20% PEG caused a significant increase of TBARS content in the seedlings of all varieties, with the most significant increase of 94% in variety Silvija (*p* < 0.001), and the lowest significant increase in varieties Pepeljuga and Anđelka, 21% (*p* < 0.001) and 45% (*p* < 0.01), respectively, compared to the control plants.

### 3.4. GSH and GSSG Content in Wheat Seedlings

Drought stress induced with 20% PEG increased the content of GSH in wheat seedlings of all varieties tested, while seedlings under 10% PEG treatment did not show a significant difference compared to the control (Figure 2C). The highest significant increase of 154% was recorded for the variety Rujana (*p* < 0.001), and the lowest increase of 39% for the variety Bubnjar (*p* < 0.05) under 20% PEG treatment relative to the control seedlings.

GSSG concentration was significantly higher in wheat seedlings of the varieties Rujana and Bubnjar under the 20% PEG treatment, while no significant changes in other varieties were observed (Figure 2D). The content of the GSSG in the variety Rujana was 102% (*p* < 0.001) and in the Bubnjar 49% (*p* < 0.01) higher compared to the control.

### 3.5. Antioxidant Enzymes Activities

**Figure 3:**
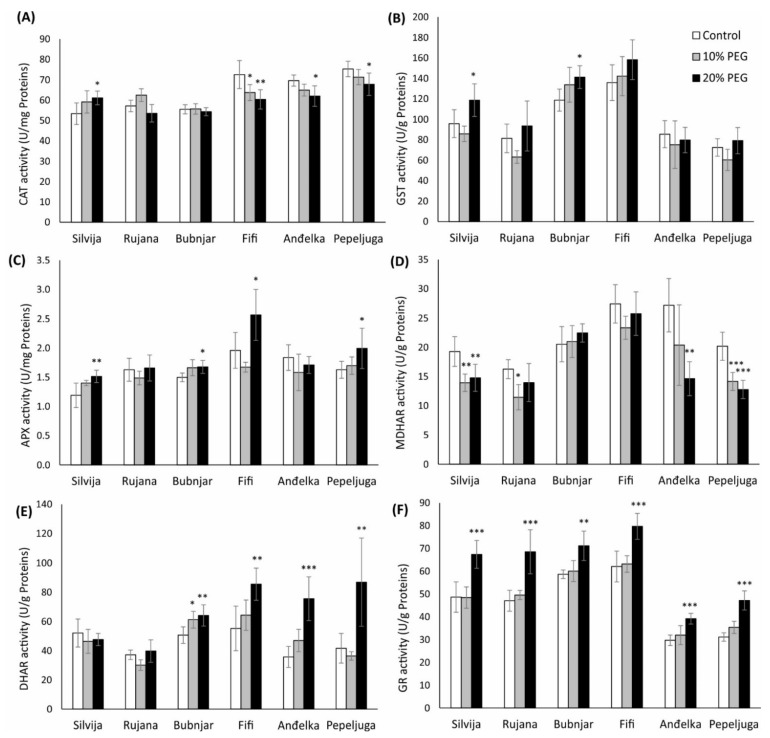
The activity of (**A**) catalase (CAT); (**B**) glutathione S-transferase (GST); (**C**) ascorbate peroxidase (APX); (**D**) monodehydroascorbate reductase (MDHAR); (**E**) dehydroascorbate reductase (DHAR); and (**F**) glutathione reductase (GR) in wheat seedlings of six Croatian wheat varieties (Silvija, Rujana, Bubnjar, Fifi, Anđelka, and Pepeljuga) under 10 and 20% PEG treatment. Expression data are presented as means of three independent biological replicates, and the error bars indicate standard deviations. Differences among treatments in each variety separately were assessed by a one-way analysis of variance (ANOVA), followed by the Dunnett post hoc test. The Dunnett test was performed at a significance level of 5, 1, and 0.1% (* *p* < 0.05, ** *p* < 0.01, *** *p* < 0.001).

CAT activity was significantly increased only in seedlings of the variety Silvija after the treatment with 20% PEG (*p* < 0.05), while the same treatment significantly decreased the CAT activity in seedlings of varieties Fifi, Pepeljuga, and Anđelka (Figure 3A). Furthermore, CAT activity in variety Fifi was significantly decreased even after the treatment with 10% PEG (*p* < 0.05).

GST activity was significantly increased in wheat seedlings of the variety Silvija and Bubnjar treated with 20% PEG (*p* < 0.05) compared to control (Figure 3B).

APX activity was significantly increased in wheat seedlings of Silvija, Bubnjar, Fifi, and Pepeljuga under 20% PEG (Figure 3C). Thus, the largest increase in APX activity of 31% was recorded in the variety Fifi, followed by an increase of 27, 22, and 12%, respectively, in the varieties Silvija, Pepeljuga, and Bubnjar, compared to the control group.

Drought stress induced with 20% PEG significantly reduced MDHAR activity in varieties Anđelka, Pepeljuga, and Silvija (Figure 3D). The most significant reductions, of 46% (*p* < 0.01) and 37% (*p* < 0.001), were observed in varieties Anđelka and Pepeljuga, respectively. Treatment with 10% PEG significantly reduced enzyme activity in seedlings of Silvija (28%), Rujana (30%), and Pepeljuga (30%) relative to the control. Under both treatments, no significant changes in MDHAR activities were observed for Bubnjar and Fifi varieties.

DHAR activity was significantly increased in wheat seedlings of most varieties under the 20% PEG treatment (Figure 3E). Variety Anđelka had the most significant increase in enzyme activity of 111% (*p* < 0.001), followed by varieties Pepeljuga (109%), Fifi (55%), and Bubnjar (27%), relative to the control. Treatment with 10% PEG significantly induced DHAR activity only in variety Bubnjar, 21% relative to the control seedlings. No significant changes in DHAR activity for both treatments with PEG were detected for the varieties Silvija and Rujana.

Treatment with 20% PEG induced a significant GR activity in wheat seedlings of all investigated varieties compared to the control (Figure 3F). The most significant increase in GR activity of 52% (*p* < 0.001) was recorded for the variety Pepeljuga, followed by variety Rujana (46%), Silvija (38%), Anđelka (32%), Fifi (28%), and Bubnjar (21%), relative to control.

### 3.6. Genes Relative Expression Levels

**Figure 4:**
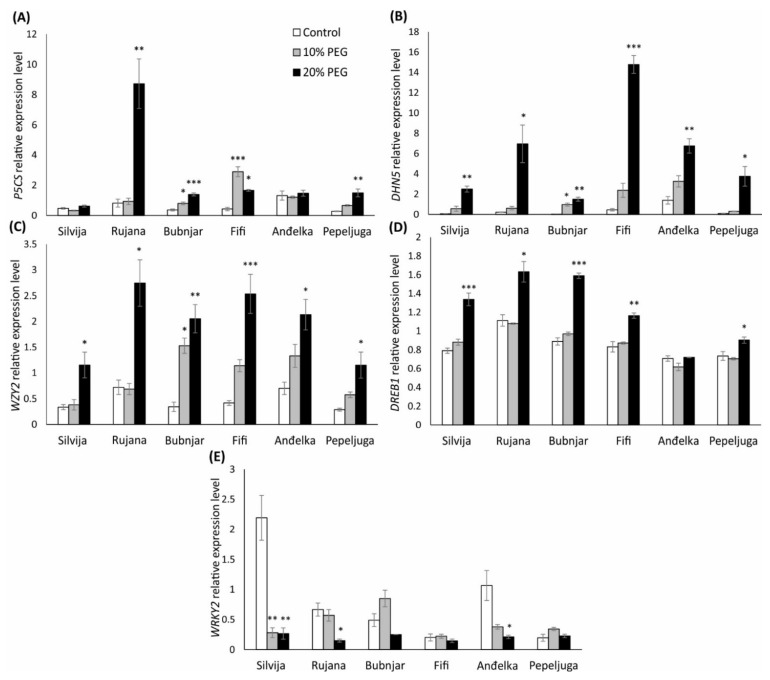
Relative expression levels of *P5CS* (**A**), *DHN5* (**B**), *WZY2* (**C**), *DREB1* (**D**), and *WRKY2* (**E**) in wheat seedlings of six Croatian wheat varieties (Silvija, Rujana, Bubnjar, Fifi, Anđelka, and Pepeljuga) under 10 and 20% PEG treatment. Expression data are presented as means of three independent biological replicates, and the error bars indicate standard deviations. Differences among treatments in each variety separately were assessed by a one-way analysis of variance (ANOVA), followed by the Dunnett post hoc test. The Dunnett test was performed at a significance level of 5, 1 and 0.1% (* *p* < 0.05, ** *p* < 0.01, *** *p* < 0.001).

The expression of the P5CS gene encoding pyrroline-5-carboxylate synthetase was upregulated due to the treatment with 20% PEG in wheat seedlings of the variety Rujana, Bubnjar, Fifi, and Pepeljuga (Figure 4A). Treatment with 10% PEG induced expression of the genes in seedlings of the varieties Bubnjar and FiFi, which was in Fifi more pronounced than expression in seedlings treated with 20% PEG. Compared to the control, no significant changes in gene expression were observed for the varieties Silvija and Anđelka.

PEG-induced drought stress upregulated the expression of the genes encoding dehydrin proteins (DHN5 and WZY2) in all varieties’ wheat seedlings in a concentration-dependent manner (Figure 4B,C). Although the genes expressions under both treatments were higher in all varieties compared to the control, a significant increase was recorded only upon the treatment with 20% PEG, with the exception of the variety Bubnjar where both treatments caused a significant increase in the expression of both genes (DHN5 and WZY2) encoding dehydrins. The increase in the relative expression of the DHN5 gene was very high, ranging from 4-fold in wheat seedlings of variety Anđelka to the 51-fold in variety Bubnjar, relative to the control. The highest expression of the WZY2 gene was recorded in seedlings of varieties Bubnjar and Fifi, 5-fold higher relative to the control. In contrast, the lowest expression was observed in seedlings of Silvija and Anđelka, with approximately 2-fold higher expression than the control.

The expression of the DREB1 gene was induced by 20% PEG treatment in wheat seedlings of most varieties tested, while variety Anđelka did not show a difference in gene expression relative to the control (Figure 4D). The highest expression of the DREB1 gene was recorded in variety Bubnjar (78%, *p* < 0.001), followed by varieties Silvija (69%, *p* < 0.001), Rujana (47%, *p* < 0.01), Fifi (40%, *p* < 0.05), and Pepeljuga (23%, *p* < 0.05).

Treatments with both PEG concentrations significantly reduced the expression of the WRKY2 gene in wheat seedlings of the variety Silvija (Figure 4E). The reduction was 87 (*p* < 0.05) and 88% (*p* < 0.01) for 10 and 20% PEG, respectively, relative to the control. Treatment with 20% PEG also significantly reduced the expression of this gene in wheat seedlings of the varieties Rujana and Anđelka, 78 and 80%, relative to the control. No significant differences have occurred in the Bubnjar, Fifi, and Pepeljuga in both treatments relative to the control.

## 4. Discussion

Wheat seedlings’ response to drought stress depended on the variety and the severity of drought stress, and it was more pronounced at the drought stress induced by 20% PEG. PEG-induced drought stress affected seed germination and morpho-physiological traits of seedlings (RWC, root and shoot length and biomass) in a concentration-dependent manner. In order to tolerate water-deficit conditions, plants adapt their morpho-physiological traits that have been considered important criteria for characterizing drought tolerant and susceptible varieties [68,69,70,71]. Germination energy was decreased in all varieties under the severe drought stress induced by the 20% PEG, while the highest reduction was observed in the variety Silvija. Seed germination is the first most sensitive stage in plant development that could be significantly affected during water deficit conditions and, therefore, compromise seedling establishment [72,73,74]. Our results are in accordance with other studies that showed a reduction of seed germination under severe drought stress [13,75]. The RWC, an indicator of water status in plants, is mainly reduced under water-deficit conditions due to impaired root water absorption [76]. The RWC has been reported as a good indicator of drought stress tolerance in leaves and could be used for the selection of drought-tolerant wheat genotypes [69,71,77]. In our study, investigated varieties expressed very similar RWC responses under applied drought stress. Both PEG treatments reduced the leaf RWC concentration-dependent, while a significant decrease was observed under severe osmotic stress in most varieties. The exception was variety Silvija where no significant difference was observed under both treatments. Despite its application in the evaluation of drought tolerance [69,71,77], RWC could not be served in this study for the selection of resistant varieties.

As indicated by shoot length and biomass reduction, severe drought stress adversely affected shoot growth in all varieties. The reduction in shoot growth was the most pronounced in varieties Silvija and Fifi, while the lowest reduction was observed for variety Bubnjar, followed by Pepeljuga and Anđelka. A significant reduction of shoot growth and biomass accumulation under water-deficit conditions was also observed in other studies conducted on wheat [13,75,78]. Reduced shoot growth is correlated with reduced leaf transpiration and evaporation caused by water deficit. In plants, water deficit induces abscisic acid (ABA) root-to-leaf signaling, promoted by soil drying through the respiration system, and the main target of this signaling is the closure of stomata [15,79]. This stomatal closure will then limit leaf conductance and gas diffusion, thus limiting the photosynthetic rate and thereby shoot growth [80].

Unlike the shoot growth, applied drought stress significantly improved root growth, as seen from increased root biomass and length. Root length was increased in most varieties under the moderate stress treatment (10% PEG), while in Bubnjar it was increased even under severe stress, suggesting its tolerance to drought stress. Roots biomass increased in almost all varieties under both PEG-treatments, except the variety Silvija where root biomass and root length were reduced under severe stress (20% PEG). The highest increase in root length and biomass was observed for the variety Bubnjar, followed by Pepeljuga and Anđelka. Induction of ABA accumulation by water-deficit conditions induces the expression of transcription factors that downregulates genes involved in cytokinins biosynthesis and signaling and also upregulates cytokinin-degrading genes (*CKX*) [81]. Cytokinins are negative regulators of root growth whose reduction results in decreased shoot:root ratio (reduced shoot and enhanced root growth), important for plants’ adaptation to drought conditions [82,83]. Improved root growth enable plants to absorb water from deeper soil layer in water-deficit conditions [84,85]. Increased root elongation could be a consequence of larger root meristems formation as cytokinins control the exit of dividing cells from the root meristem [86]. On the other hand, cytokinins are required for the shoot growth and their reduction results in reduced shoot growth in order to save limited resources and the reallocation of the resources for root growth [82,87]. In a study conducted by Bayoumi et al. [77] on wheat, PEG-induced reduction in the shoot and root biomass and coleoptiles length was more pronounced in drought susceptible than tolerant genotypes. Based on these morpho-physiological traits in current research, the variety Bubnjar could be distinguished as the most drought-tolerant variety. Variety Bubnjar showed the lowest reduction in shoot growth and the highest increase in root growth under both stress treatments. Furthermore, considering morpho-physiological traits, the impact of drought stress was the most pronounced in the variety Silvija followed by the variety Fifi. Variety Silvija was found to be the most susceptible among the other experimental varieties showing the highest reduction in germination energy, shoot length and biomass, and root length and biomass.

As an indicator of oxidative stress, lipid peroxidation levels were measured. Lipid peroxidation is a consequence of increased ROS production in plant cells under water-deficit conditions [88]. Both drought stress levels induced lipid peroxidation in seedlings of most varieties in a concentration-dependent manner. These results are consistent with the results obtained by Chakraborty and Pradhan [89], where lipid peroxidation was induced by drought stress in wheat varieties tested, with a higher increase in susceptible varieties. Lipid peroxidation is also an important biomarker of the plants’ susceptibility to stress conditions [90,91]. Abid et al. [92] showed higher lipid peroxidation levels associated with higher ROS content in the sensitive variety. In our study, the most pronounced increase of lipid peroxidation was observed in the variety Silvija, indicating more cellular damage than in other varieties and confirming its sensitivity to drought stress. On the other hand, the lowest lipid peroxidation was shown for the varieties Anđelka and Pepeljuga, indicating a stronger antioxidative response in these varieties. In addition, to better performance in morpho-physiological traits compared to other varieties, lower lipid peroxidation levels in wheat seedlings of Pepeljuga and Anđelka distinguish these varieties as drought-tolerant to some extent.

The antioxidant defense system maintains a balance between ROS production and scavenging in plant cells [93,94,95]. Antioxidative status in wheat seedlings was determined by measuring glutathione contents (GSH and GSSG) and activities of antioxidant enzymes and enzymes of the AsA-GSH pathway (CAT, GST, APX, MDHAR, DHAR, and GR). Changes in enzyme activities were dependent on the variety and the intensity of the drought stress. The most significant role in the antioxidative response of wheat seedlings to drought stress had the AsA-GSH pathway. Numerous studies revealed that enhanced activity of the AsA-GSH pathway conferred better stress tolerance [20], while increased pool size of tGSH in wheat flag leaves implicates its role in drought stress [96]. To overcome the effect of drought stress, seedlings of all varieties displayed significantly higher GSH content under severe drought stress. By participating in direct or indirect ROS neutralization in plant cells, GSH is oxidized to GSSG form reduced back to GSH by the GR enzyme [23]. In seedlings of varieties Rujana and Bubnjar, GSSG concentrations were significantly higher under severe drought stress suggesting higher depletion and lower recycling of GSH under the applied stress. Despite the increased GR activity in these two varieties, GSH was not recycled sufficiently. Higher GST, APX, and DHAR activities in seedlings of variety Bubnjar implied enhanced GSH depletion. Unlike variety Bubnjar, higher concentrations of GSSG in seedlings of variety Rujana, despite the increased GR activity, suggested the involvement of GSH in other detoxication pathways that were not analyzed in this study, such as GPX activity, glyoxalase system, and phytochelatin synthesis [97]. Variety-specific effect on the GSH pool under water deficit conditions in wheat seedlings was found by Gietler et al. [98]. Their experiment showed that the GSH and tGSH pools were higher in drought-tolerant wheat seedlings.

The primary enzymatic component of the AsA-GSH pathway that catalyzes the detoxication of H_2_O_2_ using AsA as an electron donor is APX [61]. APX activity was increased in wheat seedlings of Silvija, Bubnjar, Fifi, and Pepeljuga under 20% PEG. In our study, severe drought stress reduced MDHAR activity in varieties Anđelka, Pepeljuga, and Silvija, while treatment with 10% PEG reduced enzyme activity in seedlings of Silvija, Rujana, and Pepeljuga. As a component of the AsA-GSH pathway, MDHAR is responsible for the reduction of monodehydroascorbate to AsA using NAD(P)H as an electron donor [21]. In case of decreased MDHAR activities, monodehydroascorbate could not be converted to AsA, and dehydroascorbate will be produced. Therefore, AsA is recycled by the DHAR that catalyzes the reduction of the dehydroascorbate to AsA using GSH as a reductant. In addition to MDHAR, DHAR has an important role in maintaining the AsA pool in the plant cells [21]. In our study, DHAR activity was significantly increased in wheat seedlings of most varieties under the 20% PEG treatment, while the most significant increase was observed in varieties Anđelka and Pepeljuga. Decreases in MDHAR and DHAR activity were not correlated in our study. In the AsA-GSH pathway, GR is responsible for GSH regeneration using NADPH as a reducing agent [99]. In a study conducted by Chakraborty and Pradhan [89], GR activity showed to be the most important in conferring wheat drought tolerance. Severe drought stress induced GR activity in wheat seedlings of all investigated varieties. The most significant increase in GR activity was recorded for the variety Pepeljuga. Lascano et al. [100] showed that drought-tolerant wheat varieties had increased APX and GR activities and higher tGSH content only under in vitro osmotic stress conditions, while the same response was omitted in drought field conditions. This paper confers different responses of AsA-GSH pathway antioxidative enzymes of the same wheat varieties in different drought stress conditions mainly related to the different magnitude of stress in the studies of water deficit. On the other hand, alternative water deficit protective systems, like drought-related increase in energy dissipation related to zeaxanthin [96,101], could decrease the oxidative load on the AsA-GSH pathway, thus changing its response.

Antioxidant enzymes, GST and CAT, showed less sensitivity to drought stress, and their different regulation in varieties suggested genotype-specific responses. In addition to APX, CAT is one of the major enzymatic scavengers for detoxifying H_2_O_2_. CAT activity was increased only in seedlings of the variety Silvija under severe stress. Reduced CAT activity in seedlings of varieties Fifi, Pepeljuga, and Anđelka under severe stress was negatively correlated to lipid peroxidation levels, suggesting that the absence of CAT induction resulted in increased lipid peroxidation in these varieties. Other varieties did not show a similar pattern. GST are enzymes that catalyze the conjugation of GSH to a variety of hydrophobic, electrophilic, and usually cytotoxic substrates, as well as the conversion of H_2_O_2_ at the expense of GSH, thereby producing GSSG [102]. Some GST isoforms have glutathione peroxidase activities, which catalyze the reduction of the toxic lipid peroxidation products, thus playing an important role in the maintenance of the membrane [103]. GST activity was increased only in wheat seedlings of varieties Silvija and Bubnjar treated with 20% PEG. Previous investigations about the role of GST in drought stress are relatively inconsistent. Galle et al. [104] found that GST activity was induced by osmotic stress in moderately drought tolerant and resistant wheat varieties. Moreover, Xu et al. [105] showed that transgenic *Arabidopsis* plants expressing tomato GST enhanced drought stress resistance. Conversely, Chen et al. [106] proposed a negative role of one GST isoform (AtGSTU17) as a component of stress-mediated signal transduction pathways in adaptive responses to drought. Namely, *Arabidopsis atgstu17* mutated plants accumulated higher levels of GSH and ABA, better development of primary and lateral root systems.

Antioxidative response in plant cells under abiotic stress conditions is generally increased and correlates with cell protection and plant tolerance [107]. In a study conducted by Abid et al. [92], the tolerant wheat varieties exhibited higher antioxidant enzyme activities and lover lipid peroxidation under drought conditions compared to susceptible varieties. Enhanced antioxidant enzyme activities contribute to drought tolerance by decreasing oxidative damage [92]. Higher antioxidant enzyme activities have been reported to improve stress tolerance [108]. However, previous studies showed different and contradictory results [17]. The discrepancies in the results between different studies could be related to plant age, metabolism, tolerance to stress, duration, and the intensity of applied stress [17]. Additionally, the absence of a linear correlation between some results in our study confirmed the complexity of tolerance mechanisms in plants due to the involvement of different enzymes and genes.

In dehydration conditions, plant cells accumulate osmotically active compounds that, through osmotic adjustment, keep the main physiological functions of the cell [14]. This study analyzed the accumulation of proline as one of the main components of osmotic adjustment. In most varieties, both drought stress levels induced proline accumulation in wheat seedlings. These results are in agreement with the study of Abid et al. [92], who found an accumulation of proline during drought stress at tillering and jointing stages in wheat. Furthermore, in the study of Chakraborty and Pradhan [89], an increase in proline content was observed in the leaves of four wheat varieties exposed to drought, with higher accumulation recorded in drought-tolerant varieties. Other studies also showed that wheat’s proline accumulation contributes to increased osmotic stress tolerance [29,30,109]. In addition to osmoregulation, proline has a role in stabilizing protein and cell membrane structures and mitigating oxidative damage due to scavenging ROS [110]. This protective antioxidant role of proline is also connected with decreasing the TBARS levels, although there was no correlation between proline and TBARS content in our study. Proline accumulation was also recognized as a valuable drought tolerance indicator and could be used as a selection criterion in a wheat breeding program [30,77]. Previous studies reported higher proline accumulation under stress conditions in drought-tolerant varieties than drought-sensitive varieties [29,111]. The highest accumulation of proline was observed in seedlings of Bubnjar, with a 4-fold and 2-fold increase under 10 and 20% PEG treatment, respectively, confirming its tolerance to stress. On the contrary, the variety Silvija showed the lowest proline accumulation, consistent with its sensitivity to drought stress.

In this study, expression patterns of drought-responsive gene encoding P5CS *(P5CS*), the key enzyme in proline biosynthesis, and genes encoding dehydrins (*DHN5* and *WZY2*) were analyzed. The increase in the relative expression of the analyzed stress-responsive genes, *P5CS*, *WZY2,* and especially *DHN5*, was very high and mainly upregulated under water-deficit conditions, while their expression under the control conditions was shallow. The expression of the *P5CS* gene was upregulated under severe stress (20% PEG) in wheat seedlings of varieties Rujana, Bubnjar, Fifi, and Pepeljuga, while 10% PEG induced expression of the gene only in seedlings of varieties Bubnjar and FiFi. Upregulation of *P5CS* under PEG-induced osmotic stress was also demonstrated by Ma et al. [112]. They showed that overexpression of *TaP5CS* in transgenic *Arabidopsis* plants increased proline content and decreased lipid peroxidation under osmotic stress. In our study, the expression of *P5CS* was mostly correlated with the accumulation of proline in wheat seedlings. Many studies have previously reported a strong correlation between increased P5CR enzyme activity or transcript levels and proline accumulation [29,113], leading to increased stress tolerance [33]. The absence of correlation between proline content and the expression of *P5CS* was observed in variety Anđelka under 10% PEG treatment. This discrepancy could be due to increased protein degradation and reduced proline catabolism in the plant cell under stress [109,114]. In addition to induced proline synthesis, proline accumulation in plant cells could also be a consequence of the inactivation of proline degradation by proline dehydrogenase and pyrroline-5-carboxylate dehydrogenase [114,115]. In the study of Abid et al. [92], a correlation between decreased protein content and increased proline and other amino acid concentrations was obtained.

*DHN5* gene has been previously identified by Brini et al. [116]. In their study, *Brini* et al. [116] showed that expression of the *DHN5* gene is induced during embryogenesis, salt stress, and by ABA in vegetative tissues. In a study conducted by Wang et al. [117], the expression of the *DHN17* (the same as *DHN5*—accession no. AY619566) was also induced in leaves and roots of wheat seedlings treated with ABA, suggesting regulation by the ABA signal pathway. In transgenic studies, overexpression of *TtDHN5* enhanced tolerance to osmotic and salt stress in transgenic *Arabidopsis* plants [36,37]. Under osmotic stress, transgenic plants exhibited better growth and higher proline accumulation than wild-type plants. Saibi et al. [36] showed that overexpression of *TtDHN5* leads to salinity tolerance through proline and antioxidant metabolism regulation. In their study, *TtDHN5* enhances P5CS activity in the transgenic *Arabidopsis* plants accompanied by proline accumulation. Furthermore, the activities of antioxidant enzymes (SOD, CAT, and POD) are increased in transgenic plants under stress conditions compared to wild type [36]. In transcriptome profiling of *DHN5*-overexpressing transgenic *Arabidopsis* plants, Brini et al. [38] identified different upregulated genes, including the *MDHAR* gene important for AsA recycling in AsA-GSH pathways. They found enhanced tolerance to oxidative stress caused by H_2_O_2_. In our study, the importance of the AsA-GSH pathway in stress response was revealed, as it was upregulated, but no correlation between expression of *DHN5* and activity of MDHAR enzyme was observed. On the contrary, the activity of this enzyme was mostly reduced under the applied drought stress.

Dehydrin *WZY2* gene was isolated from a drought-induced cDNA library of wheat and identified as a drought-stress responsive gene [118,119,120]. In their study, Liu et al. [121] elucidate the regulation of *WZY2* expression using different approaches. The expression of the *WZY2* is positively regulated by the interaction of the bHLH transcription factor (*TabHLH49*) with the *WZY2* promotor, thus improving the drought stress resistance of wheat. Our study observed no linear correlation between investigated transcription factors and *WZY2* gene expression. Liu et al. [122] showed that WZY2 could have an important role in the ABA signaling pathway through interaction with protein phosphatase 2C, a key protein in the ABA signaling pathway to regulate stress-responsive gene expression in wheat. Transgenic studies revealed that transgenic RNAi (*WZY2*) wheat exhibited lower RWC, antioxidative enzyme activity, and increased lipid peroxidation than wild-type wheat under osmotic stress. On the other hand, overexpression of the *TaWZY2* in *Arabidopsis* plants showed a significant increase in tolerance to drought stress [123].

In order to understand molecular mechanisms of tolerance to drought stress in seedlings of different wheat varieties, genes encoding transcription factors (*WRKY2* and *DREB1*) that regulate the expression of stress-responsive genes were analyzed. The expression of the *DREB1* gene was induced under severe drought stress in wheat seedlings of most varieties tested. The *DREB1* gene was previously isolated from a drought-induced cDNA library of wheat and identified by Shen et al. [46]. The role of DREB1 transcription factors in wheat under water-deficit conditions is evident from its upregulation in response to drought stress, salinity, and ABA [46,47]. According to Kurahashi et al. [47], more drought-tolerant wheat varieties accumulate more *DREB1* gene transcripts under water-deficit conditions than susceptible varieties. Yousfi et al. [124] revealed induction of *DREB1* in durum wheat under salinity and drought stress, with tolerant genotypes exhibiting lower expression than susceptible ones. In our study, the highest expression of the *DREB1* gene was observed in seedlings of the variety Bubnjar, distinguished as drought-tolerant. Overexpression of *AtDREB1A* gen in transgenic wheat enhanced drought tolerance, indicating its role in adaptation to water-deficit stress [125]. In a study by Noor et al. [126], overexpression of the *AtDREB1* gene in wheat under drought and salinity stress increased RWC, proline, and other metabolites content in transgenic wheat compared to wild-type.

In this study, we analyzed gene expression of the WRKY2 transcription factor, a member of group II consisting of one WRKY domain with a C2HC zink-finger motif [42]. Previously studies showed that overexpression of the *TaWRKY2* gene in transgenic wheat seedlings and transgenic *Arabidopsis* plants enhanced tolerance to drought stress, as evidenced by improved morpho-physiological traits compared to wild-type plants [52,54]. Furthermore, the higher contents of proline and other metabolites provide protection from oxidative stress and osmotic damage. Due to its role in drought tolerance, Niu et al. [54] suggested the *WRKY2* gene as a good candidate for improved drought tolerance of wheat varieties using transgenic technology. Multiple regulatory cis-elements were identified in the promoter region of the *WRKY2* gene implying its regulation by multiple stress conditions (drought, salt, heat, and ABA) [52]. Contrary to the results obtained in mentioned studies, in our study, the expression of the *TaWRKY2* gene was downregulated by the PEG-induced drought stress. Three varieties (Silvija, Rujana, and Anđelka) showed lower levels of *WRKY2* gene transcript. The most pronounced impact of drought stress was observed for the variety Silvija where both PEG treatments reduced gene expression. No linear correlation between the expression of *WRKY2* compared to other measured parameters was observed. Variety Silvija, with a higher degree of *WRKY2* reduction, was previously distinguished as a drought-susceptible variety based on other parameters. It could be that *WRKY2* was regulated by some transcriptional repressors that modulate plant stress responses. Such a large extent in suppression of *WRKY2* gene expression in seedlings of the variety Silvija could be related to reduced morphological traits (root and shoot length and biomass) under the drought stress treatment. In their study, Hu et al. [127] identified the *WRKY51* gene as the key factor in promoting lateral root formation through negative regulation of ethylene biosynthesis in wheat. Our results suggest that reduced *WRKY2* gene expression could impact root formation, thus affecting seedling growth of the Silvija variety under drought stress conditions. This effect was not observed in other varieties, probably due to a more extensive reduction of *WRKY2* gene expression in the variety Silvija compared to other varieties. Interestingly, in transcriptome profiling of transgenic *Arabidopsis* plants with *DHN5* overexpression, *Brini* et al. [38] showed downregulation of some WRKY transcription factors (*WRKY33* and *WRKY70*). Although *WRKY2* was mostly downregulated and *DHN5* upregulated under the drought stress conditions in our study, no linear correlation between the expression of the *DHN5* and *WRKY2* was observed.

Variety Bubnjar was distinguished as the most drought-tolerant variety. Enhanced drought resistance in Bubnjar was associated with osmotic adjustment, including the expression of genes encoding dehydrin proteins. Despite the active AsA-GSH pathway, high lipid peroxidation levels and high GSSG content in Bubnjar seedlings suggest lower antioxidative response in that variety. Varieties Anđelka and Pepeljuga showed drought tolerance to some extent, although mechanisms underlying their tolerance differed compared to the variety Bubnjar. In a variety Pepeljuga, high proline content and the expression of stress-responsive genes encoding dehydrins and *P5CS* contributed to osmotic adjustment as a mechanism of tolerance together with the activity of the AsA-GSH pathway. Tolerance strategies of variety Anđelka involved only the induction of antioxidative defense through the AsA-GSH pathway. Considering obtained data, variety Silvija was found to be drought-susceptible. In the variety Silvija, regardless of the antioxidative system induction (increased APX, GST, and CAT activity) and dehydrin genes expression, drought stress caused oxidative damage to lipids and impaired root and shoot growth. Variety Fifi revealed a high expression of the dehydrin genes, increased *P5CS* expression, and consequently proline accumulation. Reduced growth parameters could be due to the direction of the metabolism towards osmolyte and dehydrin synthesis.

## 5. Conclusions

Drought tolerance involves a complex network of different mechanisms comprising a number of proteins and genes, interactions between transcription factors and corresponding genes, and different signal transduction pathways and their mutual interactions. In the present study, different mechanisms have been shown to contribute to drought tolerance in different varieties, mainly related to osmotic adjustment and dehydrins expression. The most important role of antioxidant response was played by glutathione along with the AsA-GSH pathway. Genes encoding transcription factors were shown to be differentially regulated. The expression of *DREB1* was upregulated under severe drought stress in most varieties, while the expression of *WRKY2,* unlike the other studies, was downregulated or revealed control levels, thus requiring further and broader investigation. Identifying different mechanisms in drought stress response would advance our understanding of the complex strategies contributing to wheat tolerance in the early growth stage and could contribute to variety selection useful for further development of new drought-tolerant varieties. Furthermore, elucidation of the proteins and genes involved in biochemical and molecular mechanisms under water-deficit conditions may lead to genetic improvement of wheat using transgenic technology. Considering obtained data, variety Silvija was drought-susceptible, while variety Bubnjar showed greater drought stress tolerance, suggesting better germination potential under water deficit conditions relative to other varieties.

## Figures and Tables

**Table 1:** Oligonucleotide primers sequences.

Target Gene	GenBank Accession No.	Product Length (bp)	Forward Primer	Revers Primer
*DHN5*	AY619566	99	agaagaagggcatcatggac	ggcacctccactctcagaag
*WZY2*	KF112871	142	tcgttcgtcgtggtagtctg	atgaccttgctgtccgtagg
*P5CS*	KT868850	85	ccggtgaatggcagagtaat	ccccacggagaactttaaca
*WRKY2*	EU665425	131	ctttggcttctcctttcacg	tgctgctcttgttgctcact
*DREB1*	DQ195070	80	gttggtacccaacccaagtg	aacagaacgaagcagggcta
*actin* [67]	AK457930	215	tgaccgtatgagcaaggag	ccagacaactcgcaacttag
*ADP-ribosylati factor* [67]	XM_044502292	165	gctctccaacaacattgccaac	gcttctgcctgtcacatacgc

## Data Availability

All of the data is contained within the article.
